# Cytological Characterization and Allelism Testing of Anther Developmental Mutants Identified in a Screen of Maize Male Sterile Lines

**DOI:** 10.1534/g3.112.004465

**Published:** 2013-02-01

**Authors:** Ljudmilla Timofejeva, David S. Skibbe, Sidae Lee, Inna Golubovskaya, Rachel Wang, Lisa Harper, Virginia Walbot, William Zacheus Cande

**Affiliations:** * Department of Molecular and Cell Biology, University of California at Berkeley, California 94720; † Department of Gene Technology, Tallinn University of Technology, Tallinn 12618, Estonia; ‡ Department of Biology, Stanford University, Stanford, California 94305; § N.I. Vavilov Institute of Plant Industry, St.-Petersburg 190000, Russia; ** USDA-ARS Plant Gene Expression Center, Albany, New York 94710

**Keywords:** maize, anther development, cell fate acquisition, male sterility

## Abstract

Proper regulation of anther differentiation is crucial for producing functional pollen, and defects in or absence of any anther cell type result in male sterility. To deepen understanding of processes required to establish premeiotic cell fate and differentiation of somatic support cell layers a cytological screen of maize male-sterile mutants has been conducted which yielded 42 new mutants including 22 mutants with premeiotic cytological defects (increasing this class fivefold), 7 mutants with postmeiotic defects, and 13 mutants with irregular meiosis. Allelism tests with known and new mutants confirmed new alleles of four premeiotic developmental mutants, including two novel alleles of *msca1* and single new alleles of *ms32*, *ms8*, and *ocl4*, and two alleles of the postmeiotic *ms45*. An allelic pair of newly described mutants was found. Premeiotic mutants are now classified into four categories: anther identity defects, abnormal anther structure, locular wall defects and premature degradation of cell layers, and/or microsporocyte collapse. The range of mutant phenotypic classes is discussed in comparison with developmental genetic investigation of anther development in rice and Arabidopsis to highlight similarities and differences between grasses and eudicots and within the grasses.

Plants differ from animals because they lack a germline set aside early in embryogenesis. Instead, plant germinal cells develop *de novo* from somatic cells late in development. During vegetative growth, shoot apical meristem activity produces leaves, stems, and lateral buds while maintaining a population of stem cells at the center (Steeves and Sussex 1989). Environmental and endogenous cues trigger stem cells of apical meristem in flowering plants to switch to a floral meristem, which is entirely used for a reproductive organ formation. One of these organs is the stamen, the male reproductive structure, which is a compound organ consisting of a four-lobed anther supported by a filament connected to the floral axis.

Clonal analyses have determined that both outer (LI) and inner (L2) cell layers of the floral meristem contribute to anther morphogenesis in maize (Dawe and Freeling 1990), and anther reconstruction based on confocal microscopy has elucidated the pace and pattern of cell proliferation and enlargement to explain anther morphology and cell layer development (Kelliher and Walbot 2011). Anther lobes initially contain Layer 1-derived (L1-d) epidermal cells and Layer 2-derived (L2-d) cells. Over the course of several days, three somatic cell layers plus the premeiotic archesporial (AR) cells differentiate from the L2-d (Kelliher and Walbot 2011; Wang *et al.* 2012). Histogenesis is complete when there are four layers of somatic cells arranged in a concentric “dartboard” pattern surrounding the central AR cells (Figure 1A). Each somatic cell layer (epidermis, endothecium, middle layer, and tapetum) consists of a single cell type only and is one cell wide. Concomitant with histogenesis, anticlinal cell divisions contribute to anther growth; in maize, the central AR cells proliferate to a population of ~150 per lobe and then mature into pollen mother cells (PMCs) competent for meiosis. Without the coordinated development of these five distinctive lobe cell types, proper meiosis and pollen production cannot occur, leading to male sterility.

**Figure 1:**
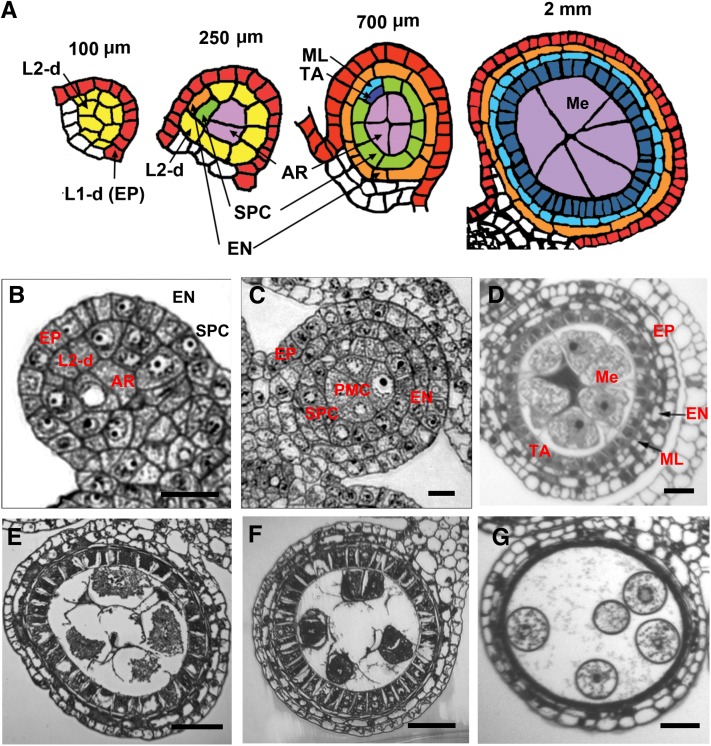
Normal anther development. (A) Illustration showing normal anther development in B73 maize. A 100-μm anther consists of the L1-derived (L1-d) epidermis (EP, red) and L2-d cells (yellow). In a 250-μm anther, the subepidermal L2-d cells start to divide periclinally generating a pair of somatic cell layers; the outer layer forms the endothecium (EN, orange) and secondary parietal cells (SPC, green). In the center of each lobe, the L2-d cells generate AR cells (purple). In a 700-μm anther, the SPC divide periclinally to form the middle layer (ML, light blue) and tapetal layer (TA, dark blue). AR (purple) cells differentiate into PMCs competent for meiosis. In a 2-mm anther, all five cell types have differentiated and meiocytes (Me, purple) have reached late prophase I. (B) Transverse section of a single anther lobe corresponding to the 250-μm illustration in (A). (C) Transverse section of a single anther lobe consisting of four cell types, EP, EN, SPC, and PMC, corresponding to the 700-μm illustration in (A). (D) Four layers of somatic cells surround the center-located early prophase meiocytes (Me). TA cells are uninucleate. (E) Tapetal cells become binucleate, middle layer flattens into a very thin layer. Meiocytes are at diakineses. Callose accumulates in the center of microsporangia. (F) PMCs are at the tetrad stage. (G) ML and TA start to degrade. Scale bar = 0.2 µm (B−D), 1 µm (E−G).

Classically, a lineage model relying on the mechanism of three sequential asymmetric cell divisions has been used to explain anther cell type specification (Davis 1966; Ma 2005). The theory was that in an immature anther lobe an L2-d hypodermal cell would divide periclinally to produce an inner sporogenous (AR) cell and an outer somatic primary parietal (transitory pluripotent) cell. Each of these cell types would proliferate, and then periclinal divisions in primary parietal cells would yield the endothecium and a secondary parietal layer. Proliferation of secondary parietal cells would be followed by a third periclinal division to generate a thin cell middle layer and a wider cell tapetal layer. This model is based on examination of transverse sections, primarily of the later stages in cell type specification. Based on new observations via confocal microscopy, AR are specified from a group of ~10 L2-d somatic cells within each anther lobe (Kelliher and Walbot 2011) rather than arising from an initial asymmetric division of a single hypodermal cell, as proposed in the lineage model. However, the model is certainly consistent for the specification of other cell layers.

Developmental genetic analysis of male-sterile mutants has contributed significantly to our understanding of the molecular mechanisms of anther development in maize, rice, and Arabidopsis. The earliest confirmed step in anther ontogeny is defined by the Arabidopsis mutant *sporocyteless/nozzle* (*spl/nzz*): the mutant lacks AR cells suggesting that the encoded transcription factor is essential for the differentiation of germinal cells from the L2-d population within lobes (Yang *et al.* 1999). The expression of SPL/NZZ has been detected as early as stamen primordia initiation and gene expression is activated by AGAMOUS (AG) (Ito *et al.* 2004), connecting SPL/NZZ to the events that specify stamen identity. Maize and rice lack obvious orthologs of *SPL/NZZ* (Xing *et al.* 2011), and it is possible that some aspects of anther ontogeny are specific to taxonomic divisions. In maize, the earliest anther developmental step is defined by *male sterile converted anther1* (*msca1*); mutants defective in this gene lack AR and anther cells differentiate as leaf cell types (Chaubal *et al.* 2003). *msca1* encodes a glutaredoxin (GRX; patent US2009/0038028A1), and a recently described rice GRX mutant *MICROSPORELESS1* (*MIL1*) also lacks AR cells (Hong *et al.* 2012).

Another instructive maize mutant is *multiple archesporial cells 1* (*mac1*). This mutant has extra AR but fewer somatic cells originated from an unknown cell type (Sheridan *et al.* 1999). MAC1 is a small, secreted protein initially expressed throughout the lobes and in part of the connective tissue before AR specification. After germinal specification, MAC1 protein levels substantially increase and localization is refined to AR cells (Wang *et al.* 2012). Interestingly, in Arabidopsis, both the *excess microsporocytes1* (*ems1*)*/extra sporogenous cells* (*exsI*) and *tapetum determinant1* (*tpd1*) mutants exhibit the *mac1* phenotypes of excess AR and fewer somatic cells. *EMS1/EXS* encodes a leucine-rich repeat receptor-like kinase (LRR-RLK) (Canales *et al.* 2002; Zhao *et al.* 2002), whereas the *TPD1* gene encodes a small, putative ligand (Yang *et al.* 2003). In rice, the *multiple sporocyte1* (*msp1*) mutant has defined an LRR receptor-like kinase required to suppress excess AR cells (Nonomura *et al.* 2003), and mutations in the *TAPETUM DETERMINANT-LIKE1A* (*TDL1A*) gene define a putative ligand with similarity to TPD1 (Zhao *et al.* 2008). Collectively, these data have been used to propose that ligand-receptor pairs coordinate communication between lobe cell layers to ensure proper proliferation and differentiation of cell types (reviewed by Zhao 2009).

In addition, many phytohormones, including auxin, gibberellins, ethylene, cytokinins, and jasmonic acid, as well as microRNAs, have been shown to regulate temporal and spatial interactions between different cell types in Arabidopsis (reviewed by Ge *et al.* 2010). To date, most of the factors identified have been inferred to act late in anther development after the division of the secondary parietal layer, suggesting that final cell fate is stabilized only late in anther ontogeny. This view is distinct from a strict cell lineage model, in which fate is irrevocably fixed at cell birth; the nature of existing mutants and the dynamic interactions among lobe cell types seem to indicate that anther cell type specification does not rely on strict lineage relationships. Instead, current insight favors the concept that cell position and communication plays a large role in somatic and germinal cell fate setting.

In Arabidopsis, differentiation of meiotic cells is inferred to require coordinated interactions with all anther wall layers and to depend on products synthesized by neighboring somatic cells (reviewed by Ma 2005; Feng and Dickinson 2010a). This is certainly true for the completion of pollen maturation, however, the fact that *mac1* AR cells mature to PMCs that start meiosis successfully without a tapetal cell layer (Sheridan *et al.* 1996) indicates that germinal cell differentiation is autonomous and independent of the presence of any normal somatic neighbors. After specification, AR cells proliferate mitotically before they switch to a meiotic cell cycle. The molecular mechanisms underlying this switch remain largely elusive. Although basic meiotic processes are evolutionarily conserved, the regulation of meiotic initiation is diverse (Pawlowski *et al.* 2007). In maize, the transition from the mitotic to meiotic cell cycle can be abolished by mutation of a single gene, *ameiotic1* (Golubovskaya *et al.* 1993, 1997). In loss-of-function *am1* mutants, cells of sporogenous morphology perform mitosis instead of meiosis; more than 25% of the anther transcriptome is aberrant at the initiation of meiosis (Nan *et al.* 2011), indicating that many processes associated with meiosis have been disrupted in both AR and somatic cells. The maize *am1* gene and a closely related gene in Arabidopsis, *SWITCH1 (SWI1)/DYAD*, encode a coiled-coil protein of unknown function (Mercier *et al.* 2001; Agashe *et al.* 2002; Pawlowski *et al.* 2009). Surprisingly, in mutants of the rice ortholog, *Osam1*, microsporocytes enter meiosis successfully but arrest at the leptotene−zygotene transition (Che *et al.* 2011), which is similar to what is observed in the maize *am1-praI* partial function mutant (Golubovskaya *et al.* 1993, 1997). Transcriptome differences between *am1-praI* and fertile sibling anthers define genes required to continue meiosis (Nan *et al.* 2011). Recently, the rice *MEIOSIS ARRESTED AT LEPTOTENE2 (MEL2)* gene encoding a novel protein with an RNA-recognition motif was found to be required for the pre-meiotic G1/S-phase transition. In *mel2* mutant anthers, most germ cells fail to enter meiosis and continue mitotic cycles while a small number of cells undergo meiosis with a significant delay (Nonomura *et al.* 2011).

Maize is highly advantageous for studying anther development and meiosis: there are hundreds of male-only florets on a tassel and anther development is highly regular. The three anthers in each floret develop largely synchronously (Hsu and Peterson 1981; Ma *et al.* 2007, 2008) and meiosis is also synchronous (Chang and Neuffer 1994). The exceptionally large size of maize anthers makes it straightforward to dissect sufficient material for biochemistry. To aid initiation of this study, there were hundreds of uncharacterized male sterile mutants resulting from the phenotypic scoring. The historic importance of male-sterility in hybrid seed production (Duvick 1965; reviewed by Laughnan and Gabay-Laughnan 1983) motivated researchers to identify and propagate male-sterile mutants. Despite the plethora of resources, however, only a handful of these male sterile mutants had been characterized cytologically and even fewer genes had been cloned. Among them *ms45* encodes a protein similar to strictosidine synthase, an enzyme involved in alkaloid biosynthesis, which is important post-meiotically (Cigan *et al.* 2001). Six genes critical for normal premeiotic development are cloned: *msca1* (patent US2009/0038028A1), *outer cell layer 4* (*ocl4*), encoding the HD-ZIP IV transcription factor (Vernoud *et al.* 2009), *mac1* (Wang *et al.* 2012), *male sterile 32* (*ms32*; J. Moon and D.S. Skibbe personal communication), *ms8* (D.S. Skibbe and V. Walbot personal communication), and *ms23* (G. Nan personal communication). Our goal was to classify hundreds of maize male-sterile mutants into premeiotic, meiotic, and postmeiotic classes, and then within the premeiotic group to further order the mutants as to time of action and severity of phenotype to define genes associated with discrete steps underlying anther locular differentiation.

## Materials and Methods

### Plant materials

A total of 244 male sterile lines were obtained from multiple sources: (1). 95 lines were obtained from the Maize Genetics Cooperation Stock Center (http://maizecoop.cropsci.uiuc.edu); (2). 67 EMS mutant lines segregating for male sterility were found in 2007 and 2008 by screening M2 populations generated by J. Hollick (Hale *et al.* 2007); (3). 23 *Mu*-insertion lines selected by I. Golubovskaya in screens of the maize-targeted mutagenesis (MTM) (Brutnell 2002) populations in 1999 and 2000, and (4) 59 *RescueMu* insertion lines carrying a transgenic *Mu1* element containing a pBluescript plasmid (Raizada *et al.* 2001; Fernandes *et al.* 2004).

### Histological analysis

From families with 20 or more plants segregating 1:1 or 3:1 for fertile to sterile, a piece of immature tassel was excised from each plant and fixed in acetic acid:ethanol 1:3 for 2 d, then stored in 70% ethanol. Approximately 2−3 wk later, the plants were scored for male sterility, and previously collected anthers from male sterile plants were examined microscopically using the aceto-carmine squash technique (Chang and Neuffer 1994). If the mutant exhibited defects in somatic or microsporocyte morphology, the fixed spikelets and/or anthers were dehydrated in a graded ethanol series, then infiltrated and embedded into low viscosity Spurr’s epoxy resin (Electron Microscopy Sciences #14300). Transverse sections approximately 1-µm thick were cut from the plastic blocks using a Reichert Ultracut E microtome, stained with 0.1% toluidine blue, and analyzed at 10× or 16× magnification under bright-field illumination.

### Genetic analysis

Mutants defective in anther development or meiosis were all recessive and were propagated by crossing ears of male sterile individuals by pollen from fertile siblings to derive families segregating 1:1 for fertile (*ms/+*) and sterile (*ms/ms*) or by self-pollination of fertile siblings to yield families segregating 3:1. Male-sterile individuals were then crossed by pollen from *ms/+* heterozygous individuals to test for allelism with the known reference mutants as well as to the panel of novel mutants (Table 1). If the genotype of the fertile plant used in a cross was unknown, as would occur in families segregating 3:1, the same plant was self-pollinated, and the progeny was scored for male sterility in parallel to the scoring of the allelism crosses.

**Table 1:** Allelism tests

Mutants	*msca1*	*ms32*	*ms8*	*ms45*	*ocl4*	*ems71924*	*tcl1*	*ms23*	*ems72063*	*ms25*	*ms26*	*mac1*	ms*6015	*csmd1*	*ms7*	*ms9*	*ms11*	*ms14*	*ems71990*	*ems72098*	ms*N22492
ms*6064	**A**	No	No	No		No	No	No	No	No	No	No									
*ems63131*	**A**	No	No	No		No	No	No	No	No	No	No									
ms*6066	No	**A**	No	No		No	No	No	No	No	No	No									
*mtm99-56*	No	No	**A**	No	No	No	No	No	No	No	No	No									
ms*-N2499	No	No	No	**A**	No		No	No	No	No	No	No		No		No	No	No			
*ems64409*	No	No	No	**A**	No	No	No	No	No	No	No	No		No	No	No	No	No	No		
*mtm99-66*	No	No	No	No	**A**	No	No	No	No	No	No	No									
*ems72032*	No	No	No	No	No	**A**	No	No	No	No	No	No	No	No	No	No	No	No	No		
*ems72063*	No	No	No	No	No	No	**A**?	**A**?	X	No	No	No	No	No	No		No	No	No		
*tcl1*	No	No	No	No	No	No	X	No	**A**?	No	No	No	No	No	No	No	No	No	No	No	No
*mtm00-06*	No	No	No	No	No	No	N0	No	No	No	No	No	No	No	No	No	No	No	No	No	
*ems63089*	No	No	No	No	No	No	No	No	No	No	No	No	No	No	No	No	No	No		No	No
ms*6015	No	No	No	No	No	No	No	No	No	No	No	No	X	No	No	No	No	No	No	No	No
ms-si*355	No	No	No	No	No	No	No	No	No	No		No	No	No	No	No	No		No		No
*csmd1*	No	No	No	No	No	No	No	No		No		No	No	X	No		No	No			
*ems71924*	No	No		No	No	X	No	No				No			No		No				
*ems71787*							No														
*ems71990*	No	No	No	No			No	No	No	No	No	No			No	No	No		X	No	
*ems72091*							No	No					No								
*ems72098*	No	No	No	No	No	No	No	No	No			No		No	No	No	No	No	No	X	
ms*N22492			No	No		No	No	No	No			No		No	No	No	No	No	No	No	X
Rescue*Mu* A60-35A	No	No			No		No	No		No	No										
*ms8*		H	X	H	No		No	H		H				No	H	H	H	H			
*ms10*	H	H	H	H	H		No	H		H	H	H	No	No	H	No	H	H	No	No	No

**A**, mutants are allelic; H, historically defined; No, mutants are not allelic; X, self.

### Search for maize orthologs

The sequences of Arabidopsis and rice genes known to be involved in anther development were used as queries for BLAST analysis against *Zea mays* Reference RNA sequences and the Nucleotide collection at NCBI (http://www.ncbi.nih.gov), MaizeGDB (http://www.maizegdb.org), and CoGe (www.genomevolution.org). A significance value of >E^1-10^ was used to identify maize orthologs or homologs as listed in Table 3. SynMap was used to identify syntenic regions between the genome of maize and rice. The gene was considered to be a syntenic ortholog when it lay within 20 genes of location predicted for an ortholog by synteny (J. Schnable and M. Freeling; www.maizegdb.org). The MUSCLE software (http://www.ebi.ac.uk/Tools/msa/muscle/) (Edgar 2004) was used to generate alignments of the predicted full-length amino acid sequences of homologs. These alignments were subsequently used to construct phylogenetic trees using www.phylogeny.fr (Dereeper *et al.* 2008).

## Results

### Landmark developmental events in fertile anthers

From an initially oval stamen tip, four anther lobes are produced nearly simultaneously, and each of these is composed of a mass of undifferentiated L2-d cells encased by a continuous epidermal layer (Figure 1A). As anther development progresses, the first internal differentiation event is specification of the centrally located AR cells. They are recognizable by their location and large size with a prominent nucleus and nucleolus, bordered by smaller L2-d cells (Figure 1B). The L2-d cells sandwiched between the epidermis and AR cells divide periclinally to form the subepidermal endothecium and the secondary parietal cells surrounding the AR (Figure 1C). During these cell specification events the entire anther doubles in length and increases in girth fueled by anticlinal cell divisions in the epidermis and L2-d population; cell division continues at a rapid pace for several days (Kelliher and Walbot 2011). Finally, secondary parietal cells divide periclinally to generate the middle and tapetal layers (Figure 1D). The anther wall consequently has four layers with an overlying epidermis and three cell layers derived from the L2-d cells. These somatic cell layers are distinctive cytologically, and each consists of a single layer of cells. Confocal microscopy of inbred line W23 showed that AR cells proliferate more slowly, reaching a population of ∼150 per lobe by 1.0 mm, then over a 2- to 3-d period the AR mature to PMCs (also called premeiotic microsporocytes or meiocytes) and meiosis starts by the 1.5 mm anther length stage. Concomitantly the tapetal layer cells expand and fill with presumptive secretory materials, giving the cells a dense cytoplasm (Figure 1, D−F); this cell layer plays a pivotal role in supporting the meiocytes by secreting macromolecules and nutrients before, during, and after meiosis. Microsporocytes seem to be connected to the tapetal layer. Callose first accumulates in the center of microsporangia (Figure 1D) and eventually surrounds each microsporocyte (Figure 1E); aberrant deposition or remodeling of callose is an underlying cause of male sterility in many mutants (Wang *et al.* 2011). When microsporocytes reach the pachytene stage of meiotic prophase 1, tapetal nuclei begin to divide without cytokinesis forming binucleate cells sporadically in the cell layer ring. By the tetrad stage, all tapetal cells are binucleate (Figure 1F). The middle layer becomes thinner and almost disappears by this stage. Six days after meiosis starts, microspores are released from the tetrads, they enlarge, and multiple small vacuoles form (Figure 1G); later, these vacuoles fuse to form one large organelle. Microsporogenesis is completed with the first pollen mitosis.

### A screen to identify mutants defective in anther development

**Figure 2:**
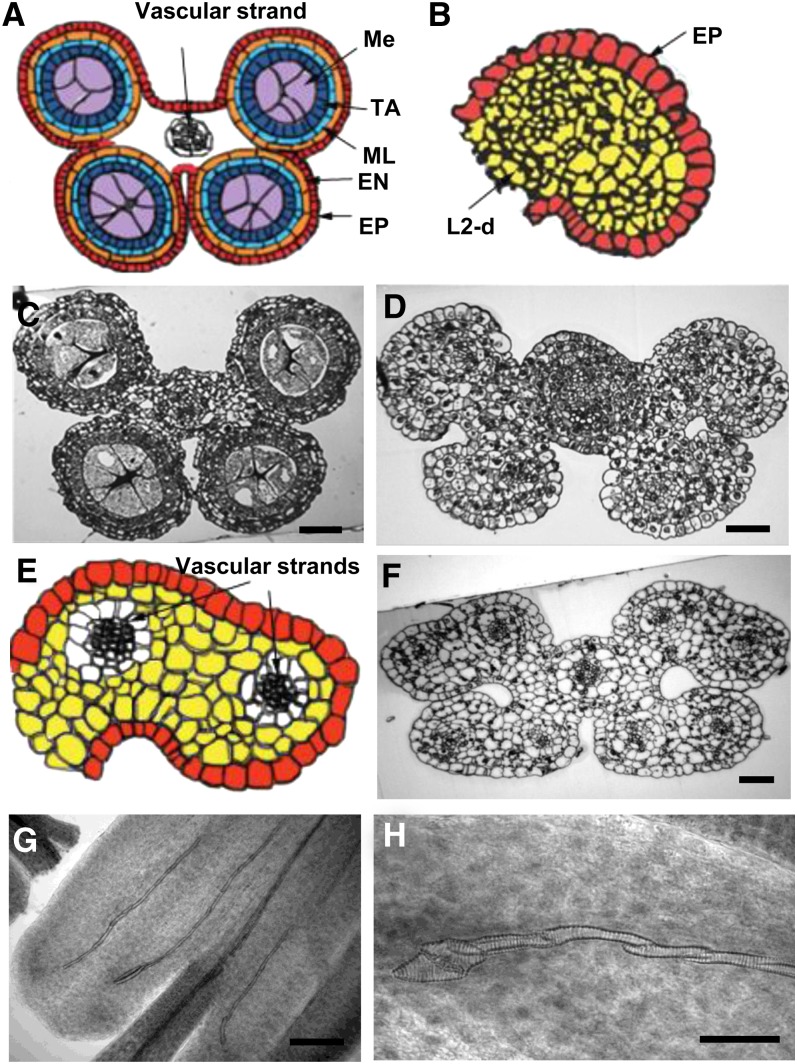
Mutants with defects in anther identity. (A) Illustration showing a transverse section of the entire anther in a fertile (normal) plant after the 700-μm stage. Centrally located meiocytes (Me, purple) are surrounded by a four-layered anther wall: EP (red), EN (orange), ML (light blue), and TA (dark blue). The vascular strand is only present in the anther filament (arrow). (B) Illustration of a single lobe of a *msca1-ems63131* anther showing undifferentiated L2-d cells (yellow) surrounded by EP (red). (C) Transverse sections of the entire anther in a fertile plant with all five cell types developed. (D) Neither anther wall layers nor PMCs are differentiated in the *msca1-ems63131* mutant anther; the lobes are filled with parenchyma-like cells at this early developmental stage. (E) Illustration of modified anther lobe with two additional vascular strands (arrows) in the *msca1-ms6064*mutant. (F) Eight nonfunctional vascular strands are present in modified anther of the *msca1-ms6064* mutant at this later stage. (G) Aceto-carmine squash of a *msca1-ms6064* anther showing vascular strands. (H) Extravascular strand appears to be nonfunctional because vessel cell walls are maintained intact between adjacent cells rather than remodeling the wall to permit lateral fluid movement. Scale bar = 1 µm (C−D and F), 0.2 µm (G), and 0.1 µm (H).

To identify genes involved in lobe cell fate decisions, we exploited the large collections of nuclear male sterile mutants: a total of 244 defined male sterile lines segregating for male sterile mutants were screened. Anthers from sterile plants were examined microscopically using the aceto-carmine squash method. While this method is typically employed to examine meiotic chromosomes, we found that it was also an excellent way to select mutants with developmental defects (Figure 2, G and H), although minor defects in lobe wall layers may not have been detected. Therefore, we have likely underestimated the yield of premeiotic mutants.

Lines that segregated plants with anthers defective in one or more cell types were classified as anther development mutants. Male-sterile plants with aberrant meiotic chromosome segregation were classified as meiotic mutants and the remaining sterile mutants with normal premeiotic and meiotic phenotype were classified as postmeiotic mutants. Altogether, 13 novel meiotic mutants and 29 anther development mutants heritable as monogenic traits were identified in the screen. The mutants classified as meiotic were both male and female sterile. All anther developmental mutants were female fertile, indicating that their defects are unlikely to be meiotic.

#### Allelism testing:

To determine whether we identified new alleles of known genes and to determine the number of new loci represented, we completed alleleism tests on nearly all new mutants, with each other, and with known mutants; a few tests are still in progress. Results are shown in Table 1. Of 29 male sterile mutants identified as anther development mutants, 7 are alleles of the previously identified anther developmental genes (two novel alleles of *msca1* and single new alleles of *ms32*, *ms8*, and *ocl4* and two alleles of *ms45*). The remaining 18 mutants fall into 16 complementation groups. Because most loci were represented by only one allele, the screen for male sterility was not saturated; more anther development mutants may still be found using this approach.

### Classification of anther developmental mutants

To better understand the developmental defects in each mutant, transverse sections were examined microscopically. This analysis allowed us to classify the 29 mutants into four groups according to their defects in anther morphology or similarity with known mutants (Table 2).

**Table 2:** Classification of mutants

Phenotype	Mutant
1. Anther identity defects	
Absence of anthers in florets	ms-si*355, *ems71990*
Anther lobe cell types fail to be specified	*msca1-ems63131*, *msca1-ms6064*
2. Anther structure defects	
Two-lobed anthers	*vlo1-ems71924*, *vlo1-ems72032*
3. Anther wall layer defects	
Undifferentiated cell layers	*ems63089*, *mtm00-06*, *tcl1*, *ems72063*
Additional periclinal division in subepidermal cell layer	*ocl4-mtm99-66*
Additional periclinal division in the middle layer	*ems72091*,
Extra cell divisions in “tapetal” layer	ms*6015, *ms32*, *ms32-ms6066*, *ms23*, *ems72063*
Multinucleate tapetal cells	*ems63265*, *ems71777*, *RescueMu-E03-23*
4. Premature layer degradation	
A. Failure to maintain anther morphology	
Meiocyte and tapetum degradation	*ms8*, *ms8-mtm99-56*, *RescueMu-A60-22b*, *ems71884*, *ems64486*
Tapetum vacuolization and degradation	*ems71787*, *RescueMu-P19-47*
Tapetal cell shrinkage and degradation	*ems71986*, *RescueMu-C17-32*, *RescueMu-A60-35A*
B. Function failure	
Lack of callose deposition	*ms10*,
Callose accumulation	*ms45-msN2499*, *ms45-ems64409*, *csmd1*, *ms8*, *ms8-mtm99-56*

#### Anther identity defects:

This group consists of four mutants with anther identity defects. Two mutants, ms-si*355 and *ems71990*, lack anthers within spikelets at the time when immature anthers normally exist (not shown). Anthers may initiate and then regress or may not initiate properly. Allelism tests showed that these mutations are not allelic to each other or to known anther developmental mutants (Table 1). Further characterization of these mutants will allow us to determine whether defects occur at the time of anther initiation or afterward during its growth.

Two other mutants, ms*6064 and *ems63131*, exhibit highly irregular anther differentiation (Figure 2, D−H). Microsporangia and all cell layers typical of the wild type anther wall fail to differentiate. Four modified lobes are characterized by an extended oval cross section, rather than the round shape typical for fertile anthers. Each aberrant lobe contains two symmetrical nonfunctional vascular strands in addition to the vascular strand continuous with the anther filament (Figure 2, E and F). In a squash specimen, these additional parallel vascular strands are not connected to the vasculature in the central connective zone of the anther (Figure 2G). Transformation of cells to vascular strands is incomplete: cell walls between cells forming vascular strand are still maintained, dividing strands to sections (Figure 2H). Multiple parenchymal cells surround the vascular strands filling the locules. The epidermal surfaces of both *ems63131* and ms*6064 mutants contain stomata; a characteristic not normally found in anthers. This suite of phenotypes resembles the *msca1* (*male sterile converted anther1*) mutant (Chaubal *et al.* 2003). Allelism tests confirmed that both newly discovered mutants are alleles of *msca1* (Table 1). Thus, we designed *ems63131* as *msca1-ems63131* and ms*6064 as *msca1-ms6064*. To date, the phenotypes of *msca1* are unique in flowering plants; the anther lobe cell types fail to be specified. The existence of this mutant strongly supports the theory that leaf cell differentiation is the default program in a lateral floral organ. Furthermore, *msca1* illustrates that overall organ shape does not depend on normal cellular composition, at least in maize anthers, as does the *tangled-1* mutant in the leaves (Smith *et al.* 1996).

#### Anther structure defects:

**Figure 3:**
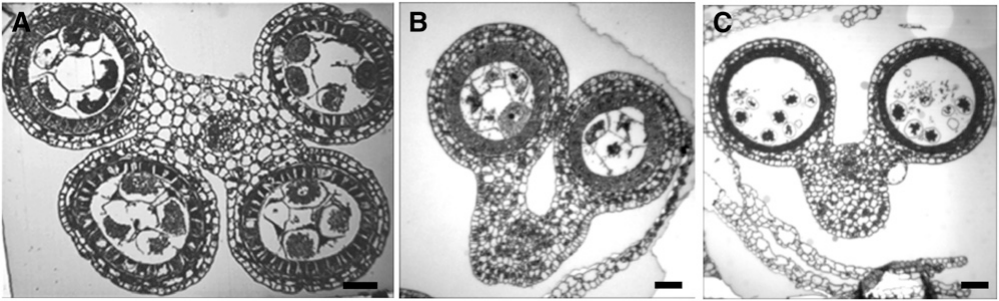
Anther structure defects. (A) Transverse section of a normal four-lobed anther in a fertile plant. (B) Two-lobed anther in *vlo1-ems71924* and (C) in *vlo1-ems72032*. Scale bar = 1 µm.

This group consists of two mutants: *ems71924* and *ems72032*. Both mutants contain fewer anthers per floret than wild type. A single anther, rather than three, per floret is the most common in both *ems71924* and *ems72032* florets. In addition, anthers have a reduced number of lobes. Unlike the normal bilaterally symmetrical four-lobed fertile anthers (Figure 3A), most mutant anthers have a two-lobed structure: the abaxial lobes are developed properly with all wall layers, while adaxial lobes fail to form (Figure 3, B and C). Although meiosis seems to progress regularly in the abaxial locules, microspores degenerate and mutant plants exhibit complete sterility. Allelism tests showed that *ems72032* and *ems71924* are allelic; however, they are not allelic to any other mutants (Table 1). The allelic pair designated *variable lobes1-ems71924* (*vlo1-ems71924*) and *vlo1-ems72032*, resembles the Arabidopsis *roxy1*, *2* double mutant in which the adaxial lobes are defective very early and later PMCs are disrupted in the abaxial lobes as well (Xing and Zachgo 2008).

#### Anther wall layer defects:

Mutants of this group fail to properly form the four layers within each anther lobe. The class is the most numerous from the screen with 12 mutants divided into two subcategories.

##### Undifferentiated cell layers:

**Figure 4:**
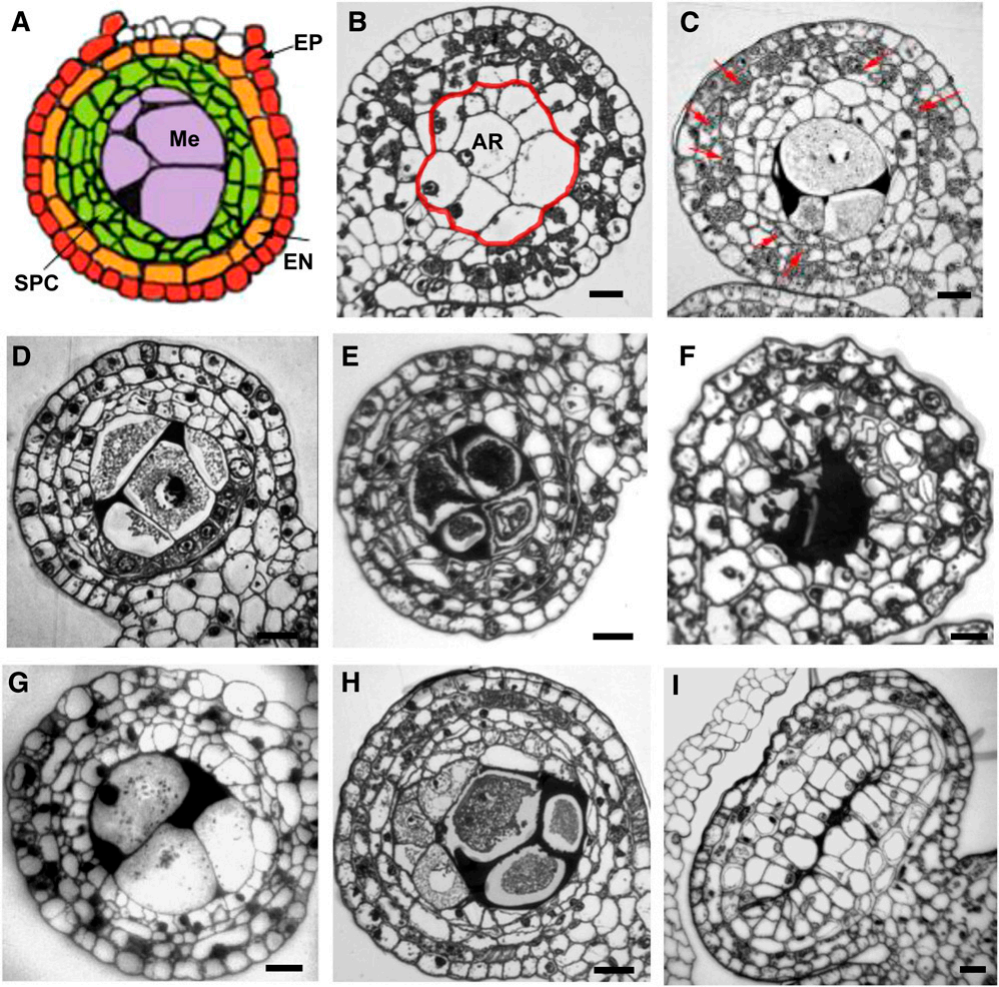
Anther wall layer defects. (A) Illustration of generalized anther wall defects demonstrating multilayered SPCs (green) between endothecium (EN, orange) and meiocytes (Me, purple) (B) Transverse section of an *ems63089* mutant anther showing undifferentiated cell layers surrounding AR cells (traced in red). Only the epidermis and a subepidermal layer are arranged in concentric layers. Neither the middle nor tapetal layer cell types differentiate. (C) Only a few meiotic cells can be observed in *ems63089* mutant anthers at later stages. Vacuolated cells of unknown origin form multiple disorganized cell layers around meiocytes. Callose starts to accumulate between meiocytes. Several subepidermal layers include cells containing substantial starch (arrows). (D) Undifferentiated cell layers surround the PMCs in *mtm00-06* anthers. Unlike normal endothecium, the subepidermal layer has no starch granules. The lobe consists of four or five layers but complete middle and tapetal layers are not observed. (E) Cells adjacent to the microsporocytes become vacuolated and disorganized. Meiocytes start to degrade before completing meiosis. (F) Meiocytes are completely degraded. Cells of all layers become vacuolated and lose their layer-specific shapes. (G) Undifferentiated cell layers in *tcl1* mutant anthers. (H) Vacuolization of cell layers adjacent to PMCs in *tcl1*. Starch granules can be observed in subepidermal layer (endothecium). (I) The five-layered anther wall in *ems72063* suggests an additional periclinal division has occurred. Scale bar = 0.2 µm

In the *ems63089* mutant, there are four or five somatic wall layers, but neither the middle nor tapetal layer cell types are observed (Figure 4, A−C). Instead, the locular volume is filled with parenchyma-like cells; these remain undifferentiated, and they do not form concentric cell layers. Only the epidermis appears unaffected by the mutation. Disorganized cells in two or three subepidermal layers contain substantial starch, a typical characteristic of the subepidermal endothecium. Because multiple starch-containing layers occur in *ems63089*, we conclude that endothecial specification is affected as well as defects in periclinal division control. The PMCs do not complete meiosis and degrade in meiotic prophase1. The *ems63089* mutant shares some similarity with the *mac1* mutant, which also fails to differentiate tapetal and middle layers. In contrast to *mac1* in which there are excess meiocytes, the number of meiocytes in *ems63089* appears to be reduced.

The novel mutant *mtm00-06*, obtained from a *Mu* transposon population, develops small transparent anthers. Neither middle nor tapetal cells differentiate (Figure 4, D−F). Instead, vacuolated cells not organized into discrete layers are observed in the anther wall. The subepidermal endothecium lacks starch granules suggesting that this layer is also functionally defective in *mtm00-06* or that anther nutritive status is very poor preventing storage of materials. Microsporocytes enter meiosis but are unable to proceed through it and degrade.

Another novel mutant *tcl1* (*tapetal cell layer1)* also fails to form coherent middle and tapetal layers, and there are extra cells between the endothecial and AR cells. Cells adjacent to microsporocytes become vacuolated (Figure 4, G−H). Despite phenotypic similarities among *tcl1*, *ems63089*, and *mtm00-06*, these three mutants are not allelic and define three loci involved in acquisition or maintenance of the differentiated state in secondary parietal cell derivatives.

##### Extra cell layers:

The *ems72063* mutant displays an additional periclinal division in tapetal initials ultimately forming a five layered anther wall (Figure 4I). Interestingly F1 progenies from crosses of heterozygous *ems72063* plants with heterozygous *tcl1* or *ms23* segregated for sterility while these two mutations were found to be not allelic (Table 1). The testing of possible additive effect of mutations is in progress.

**Figure 5:**
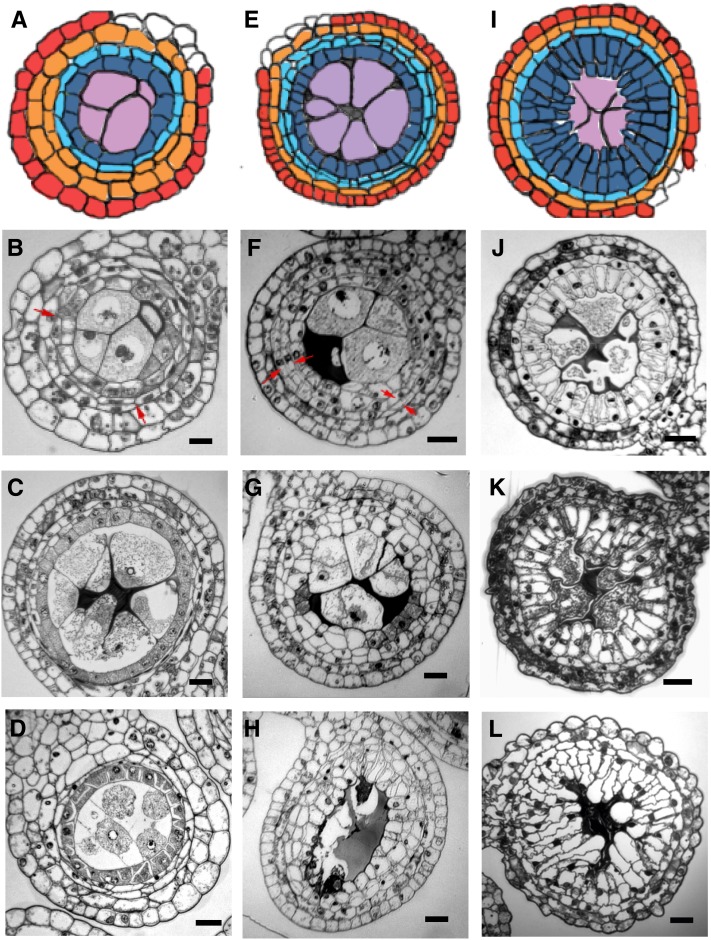
Defects in cell proliferation. (A−D) Cartoon and transverse sections of the *ocl4-mtm99-66* mutant. (A) Illustration showing an extra periclinal division of the subepidermal cell layer (orange). (B) An additional subepidermal cell layer is restricted to the outer portion of anther lobe in the *ocl4-mtm99-66* mutant (arrows). (C) Tapetal cell layer development and callose accumulation around meiocytes appears normal at this stage. (D) After meiosis, microspores are able to release from tetrads, suggesting that the anther somatic cells provide what is needed to complete meiosis. (E−H) Cartoon and transverse sections of the *ems72091* mutant. (E) Cartoon demonstrating an additional periclinal cell division in the middle layer (light blue). (F) While younger anthers appear normal, extra periclinal divisions leading to an additional cell layer can be seen in this cross section (arrows).(G) Cells in the middle and tapetal layers become vacuolated and disorganized by the tetrad stage. Microspores start to degrade. (H) Microspores are degraded and cell layers become even more disorganized. (I−L) Cartoon and transverse sections of the ms32-ms*6066 mutant. (I) Cartoon showing excess proliferation of cells in the position of the tapetal cell layer (“tapetal” cells, dark blue). (J) Uninucleate “tapetal” cells enlarge and become vacuolated. (K) Modified “tapetal” cells start to divide periclinally and protrude into the microsporocytes. (L) Extra periclinal cell divisions result in a multilayered “tapetum,” which appears to crush the meiocytes. Scale bar = 0.2 µm

An additional cell layer, restricted to the outer portion of each lobe, was apparent in the *Mu*-insertion line, *mtm99-66* (Figure 5, A−D). The phenotype of extra subepidermal cells only in the lobe overlain by epidermis resembles the phenotype described for the *ocl4* (*outer cell layer4*) mutant; these mutants define a new axis of anther organization in which subepidermal events are controlled differently depending on whether there is overlying epidermis or cells are bordered by the connective parenchyma at the center of the anther. *ocl4* encodes a HD-ZIP IV transcription factor (Vernoud *et al.* 2009), and *mtm99-66* was found to be a new allele of this locus. In the original report, the additional divisions in *ocl4* were interpreted as deriving from the endothecial layer. Markers for each cell layer are needed to clarify the origin of this ectopic partial layer caused by mutations in *ocl4* and its allele *mtm99-66* designated as *ocl4-mtm99-66*.

Extra periclinal divisions in the middle layer were detected in *ems72091* (Figure 5, E−H). Initially, all wall layers, including the tapetal layer, are formed. Later, cells of the middle layer divide periclinally to form a five-layered anther wall. By the tetrad stage, cells of both the middle and tapetal layers become vacuolated and the microsporocytes degrade. Unlike *mac1* and *ems63089* mutants, however, where regular cell layers fail to differentiate, *ems72091* is able to form all four layers but fails to maintain them in their normal differentiated state because the defective, vacuolated cells lose their layer-specific shapes (Figure 5, G and H).

In the ms*6066 mutant (Figure 5I), tapetal cell precursors fail to differentiate normally and become highly vacuolated (Figures 5J). In fertile anthers, tapetal nuclei divide without cytokinesis to form binucleate tapetal cells, a process initiated at the start of meiosis and finished by the tetrad stage. Binucleate cells were not observed in the layer adjacent to microsporocytes in ms*6066. Instead, extra periclinal cell divisions occur resulting in a multi-layered tapetum. As anther development progressed, defects became more severe. The vacuolated tapetal zone cells enlarged, eventually crushing the sporogenous cells (Figure 5, K−L). Microsporocytes enter meiosis but do not complete the meiotic division. We found ms*6066 to be allelic to *ms32* (Table 1).

**Figure 6:**
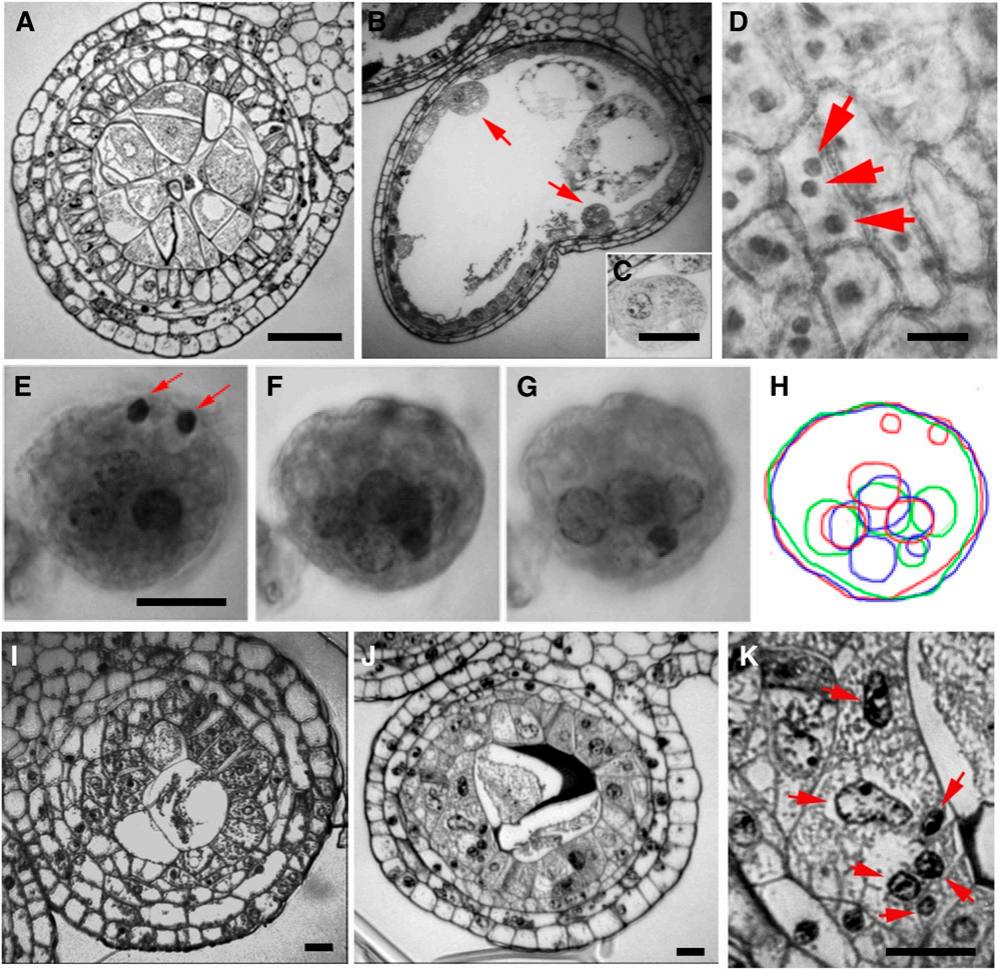
More defects in cell proliferation. (A) Additional anticlinal divisions with irregular wall placement result in extra and abnormal cells in the “tapetal” layer in the ms*6015 mutant anther. Extra microsporocytes are also present. (B−H) Cell defects in *ems63265* mutant anthers. (B) Transverse section showing several enlarged and rounded “tapetal” cells; a close-up can be seen in (C). (D) Some “tapetal” cells undergo multiple nuclear divisions without cytokinesis, forming multinucleated cells that can be observed in aceto-carmine squashes of anthers from the pachytene stage through the late microspore stage. Most microspores become multinucleate. (E−G) Images of different focal planes from a single multinucleated cell with 10 nuclei, two of them are pyknotic (arrows). (H) Illustration of the same cell with traced nuclei: from the image E in red, from the image F in blue and from the image G in green. (I) Disorganized tapetal cells in transverse section of the *ems71777* mutant anther. Some of these cells have additional nuclei. (J) Disorganized “tapetal” cells with different numbers of nuclei in *RescueMu-E03-23* mutant anther. (K) Enlarged fragment of image J showing “tapetal” cell with six nuclei (arrows).

In the ms*6015 mutant, tapetal cells undergo additional anticlinal divisions with irregular wall placement resulting in extra and abnormal cells in the “tapetal” layer (Figure 6A). Like ms*6066, binucleate tapetal cells were never found in ms*6015. Allelism tests showed that ms*6015 is not allelic to *ms32*/ ms*6066 or any known gene tested (Table 1).

Three nonallelic mutants, *ems63265*, *ems71777*, and *RescueMu-E03-23*, share several phenotypes. First, nuclei of a few tapetal cells undergo multiple divisions without cytokinesis forming huge multinucleated cells with up to eight nuclei (Figure 6, B−H). Alternatively, cell wall degradation between tapetal cells followed by fusion of several tapetal cells could result in this phenotype (Figure 6, I−K). The inward facing wall of tapetal cells is partially degraded just before meiosis; however, degradation of the lateral walls separating adjacent tapetal cells is not part of normal development. Interestingly, only a few tapetal cells become multinucleate, a feature that may reflect the intrinsic growth potential of a subset of cells (Feng and Dickinson 2010a). During tapetal ontogeny there is a period of rapid cell proliferation anticlinally, however, not all cells divide an equal number of times (Kelliher and Walbot 2011); one hypothesis to explain the presence of a few multinucleate cells is that the last few nuclear divisions occur without subsequent cytokinesis. Subsequently, microsporocytes and tapetal cells degenerate, and the middle layer cells become vacuolated (Figure 6I).

#### Premature layer degradation:

The 14 mutants in this class are divided into two subcategories (Table 2):

##### Failure to maintain anther morphology:

The 10 mutants in this class develop normal cell layers and reach an appropriate number of sporogenous cells but are not able to maintain cell identity throughout development and cells die prematurely. As development proceeds, the tapetal cells either become abnormally vacuolated and enlarged or shrink, depending on the mutation. Chromatin in tapetal nuclei undergoes irreversible condensation (pyknosis) and tapetal cells degrade. These processes impact meiotic cells, which fail to complete meiosis. It is formally possible that the fundamental defect is in the meiocytes—these cells show nuclear abnormalities and cytoplasmic shrinkage, and these events could trigger tapetal cells degradation.

**Figure 7:**
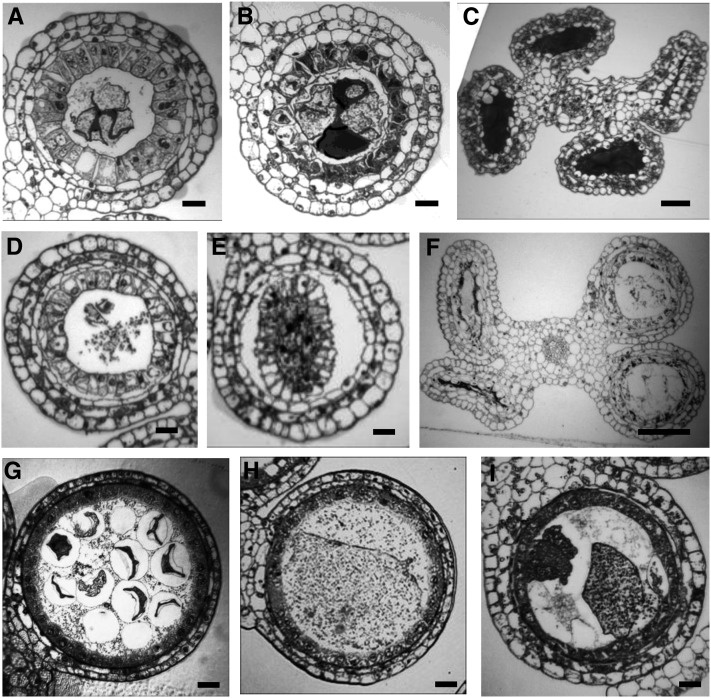
Microsporocyte and microspore degradation. (A−C) Transverse anther sections of the *ms8* mutant. (A) Meiocytes start to degrade at the tetrad stage (*ms8-ref allele)*. Binucleate tapetal cells look normal at this stage; however, cells of the middle layer start to become vacuolated. (B) Later, cells in the tapetal layer degrade. Vacuolated cells in the middle layer enlarge. Excess callose accumulates in the anther locule. (C) Transverse section of entire anther of the *ms8-mtm99-56* mutant. Meiocytes and tapetal cells are completely degraded; the remaining cell layers become vacuolated. Subsequently more callose accumulates in anther locules. (D−F) Meiocytes and tapetal cells degrade in the *RescueMu-A60-22b* transgenic anther (compare D, E, F with A, B, C, respectively). Unlike the *ms8* mutants, callose accumulation in *RescueMu-A60-22b* anther locules appears to be normal. (G−H) *ems71884* mutant. (G) After release from tetrads, microspores degrade. Note that the tapetal cell layer looks normal and microspore cell walls do not shrink. (H) At latter stages, microspores are completely degraded. (I) Microspores also degrade in the *ems64486* mutant anther. Scale bar = 0.2 µm (A−B and D−E), 1 µm (C and F)

**Figure 8:**
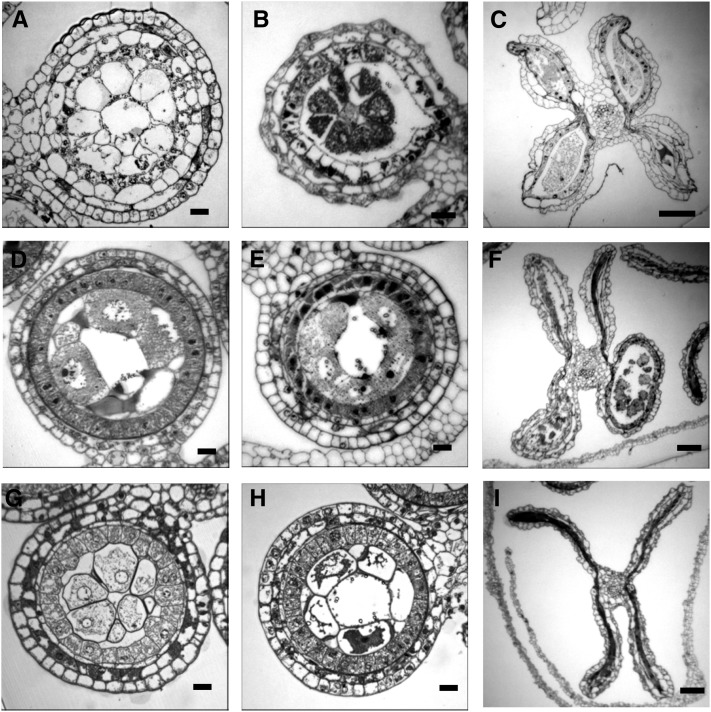
Premature anther wall layer degradation. (A) Transverse section through the *ems71787* mutant anther shows degraded cells of the tapetal layer, while cells of middle layer become highly vacuolated. (B−C) Transverse sections through the *RescueMu-P19-47* mutant anthers. (B) Cells of the endothecium and tapetum become vacuolated when microsporocytes are at the tetrad stage. (C) At latter stages, the tapetal layer lose their borders and microspores degrade completely. (D) In the *ems71986* mutant when meiocytes are in meiotic prophase, chromatin in the tapetal nuclei irreversibly condenses, their cytoplasm shrinks, and tapetal cells undergo degradation. (E−F) Transverse sections of the *RescueMu-C17-32* mutant anthers. (E) The mutant anther displays a similar phenotype: irreversible condensation of chromatin in the tapetal nuclei. (F) Degradation of anthers involves all cell layers. Anther lobes shrink. (G−I) Transverse sections of *RescueMuA60-35A* mutant anthers. (G) In *RescueMuA60-35* transgenic anthers, microspores and some tapetal cells dramatically enlarge in size. (H) Microspore degradation in anthers is not accompanied by middle layer and/or tapetal cell vacuolation. (I) Degradation of cell layers leads to a shrinkage of anther locules. Scale bar = 0.2 µm (A−B, D−E, G−H), 1 µm (C, F, and I).

In *ms8* (Figure 7, A and B) and *mtm99-56* (Figure 7C), which we found to be a new allele of *ms8* (Table 1), as well as in *RescueMu-A60-22b* (Figure 7, D−F), *ems71884* (Figure 7, G−H), and *ems64486* (Figure 7I) mutants, meiocytes collapse completely after the tetrad stage. Tapetal cells become vacuolated and subsequently degrade. Interestingly, *ms8* also exhibits several mild defects: an excess number of epidermal cells that are shorter than normal, but fewer tapetal cells that are larger than normal, and an excess callose accumulation during meiosis (Wang *et al.* 2010). Excess callose also accumulates in *ms8-mtm99-56* allelic mutant (Figure 7C). Unlike the *ms8* mutants, callose accumulation is normal in *RescueMu-A60-22b* mutant (Figure 7F). Vacuolization and premature degradation of cell layers was detected in EMS-induced mutant ems*71787* (Figure 8A) and in the *RescueMu-P19-47* transgenic line (Figures 8, B and C).

Premature degradation of tapetal cells can also occur without cell vacuolization. Chromatin in the tapetal nuclei irreversibly condenses, the cytoplasm shrinks, and cells undergo degradation in *ems71986* (Figure 8D) and in two transgenic lines: *RescueMu-C17-32* (Figure 8, E and F) and *RescueMu-A60-35A* (Figure 8, G−I). Degradation of tapetal cell layers followed by degradation of meiocytes and vice-versa suggests that there is close coordination between these two cell layers or that loss of integrity in one layer triggers general processes that result in anther abortion. Degradation of cell layers results in anther shrinkage (Figures 7, C and F and Figure 8, C, F, and I) and may be a contributing factor to growth arrest observed in male sterile mutants.

##### Functional failure:

Our definition for mutants in this subcategory is that anthers differentiate all cell layers but exhibit a functional defect that is independent of cell identity. One example is the historic mutant *ms10*, which is deficient in callose deposition. We identified new alleles of existing genes in this category, but no new loci. Two newly identified alleles of *ms45*, ms*N2499 and *ems64409*, (designated as *ms45-msN2499* and *ms45-ems64409*) display an irregular pattern of callose deposition. In fertile anthers, callose first accumulates in the center of microsporangia and eventually surrounds each microsporocyte, whereas in *ms45-msN2499*, callose deposition starts at the periphery of microsporangia. These two mutants also have slight post-meiotic defects in the tapetal layer and are completely male sterile.

### Search for maize orthologous or homologous genes matching genes in other species known to be required for proper anther development

**Figure 9:**
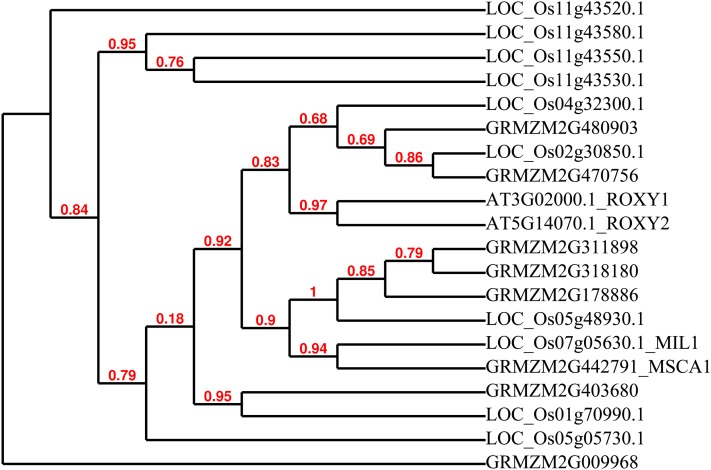
Reconstruction of phylogeny of MSCA1. *msca1* (a gene model GRMZM442791) ortholog is present in rice (Os07g05360) but is missing in Arabidopsis; the most related are Arabidopsis *ROXY2* (At5G14070) and *ROXY1* (At3G02000) encoding glutaredoxin-C8 and C7, respectively. Numbers show branch support values.

As part of our analysis of the steps in maize anther development, we were curious to determine if maize contains orthologsor homologs of genes already identified through genetic analysis as critical for anther ontogeny in rice or Arabidopsis. Despite the high conservation of anther structure in flowering plants, as highlighted previously, the grasses lack SPL/NZZ, a key regulator in dicots. To what extent will clade-specific or even species-restricted gene types contribute to anther development? BLAST analysis followed by phylogenetic analysis of the sequences of Arabidopsis and rice genes known to be involved in anther development identified maize orthologs or homologs for several key rice and/or Arabidopsis genes; as expected, some genes such as genes encoding the LRR-kinases are present in gene families in maize and suggesting multiple putative homologs (Table 3). Reconstruction of phylogeny of MSCA1 and SPL suggests that a *msca1* ortholog is missing in Arabidopsis (Figure 9) and that maize lacks the orthologous gene of Arabidopsis *SPL*.

**Table 3:** Maize genes related to rice and Arabidopsis genes involved in anther development

Gene name in *Zea mays*	Gene model	Protein encoded	Gene name in *Oryza sativa*	Gene ID	Gene name in *Arabidopsis thaliana*	Gene ID	Protein encoded
1. Genes regulating anther identity							
* si1*, *silky1*	GRMZM2G139073	SRF-type transcription factor	*OsMADS16*	Os06g0712700	*AP3*, *APETALA3*	At3g54340	MADS-box transcription factor
* zmm16*, *MADS16*	GRMZM2G110153	MADS-box transcription factor	*OsMADS2*	Os01g0883100	*PI*, *PISTILLATA*	At5g20240	MADS-box transcription factor
* zmm29*	GRMZM2G152862	SRF-type transcription factor	*OsMADS4*	Os05g34940	*PI*, *PISTILLATA*	At5g20240	MADS-box transcription factor
	GRMZM5G805387	SRF-type transcription factor	*OsMADS4*	Os05g34940	*PI*, *PISTILLATA*	At5g20240	MADS-box transcription factor
* zmm2*, *MADS2*	GRMZM2G359952	SRF-type transcription factor	*OsMADS3*	Os01g10504	*AG*, *AGAMOUS*	At4g18960	MADS-box transcription factor
* zag1*, *zea agamous homolog1*	G890RMZM2G052	SRF-type transcription factor	*OsMADS58*	Os05g11414	*AG*, *AGAMOUS*	At4g18960	MADS-box transcription factor
* zag2*, *zea agamous homolog2*	GRMZM2G160687	SRF-type transcription factor	*OsMADS13*	Os12g10540	*AGL5*, *SHP2*	At2g42830	MADS-box transcription factor
* zag3*, *zea agamous3*; *bde1*, *bearded-ear1*	GRMZM2G160565	SRF-type transcription factor	*OsMADS6*	Os02g45770	*AGL6*, *AGAMOUS-like6*	At2g45650	MADS-box transcription factor
* wus1*, *wuschel1*	GRMZM2G047448	Homeobox domain containing protein		Os04g56780	*AtWUS*, *WUSCHEL*	At2g17950	Homeodomain-like superfamily protein
* wus2*, *wuschel2*	GRMZM2G028622	Homeobox domain containing protein		Os04g56780	*AtWUS*, *WUSCHEL*	At2g17950	Homeodomain-like superfamily protein
* zfl1*, *zea floricaula/leafy1*	GRMZM2G098813	transcription factor FL		Os04g0598300	*LFY*, *LEAFY*	At5g61850	transcription factor
* zfl2*, *zea floricaula/leafy2*	GRMZM2G180190	transcription factor FL		Os04g0598300	*LFY*, *LEAFY*	At5g61850	transcription factor
					*SEP1*, *SEPALLATA1*	At5g15800	
* zmm31*, *MADS31*	GRMZM2G071620	SRF-type transcription factor	*OsMADS34*	Os03g54170	*SEP2*, *SEPALLATA2*	At3g02310	MADS-box transcription factor
* zmm24*	GRMZM2G087095	SRF-type transcription factor	*OsMADS34*	Os03g54170	*SEP2*, *SEPALLATA2*	At3g02310	MADS-box transcription factor
* zmm6*, *MADS6*	GRMZM2G159397	SRF-type transcription factor	*OsMADS7*	Os08g41950	*SEP3*, *SEPALLATA3*	At1g24260	MADS-box transcription factor
* td1*, *thick tassel dwarf1*	GRMZM2G300133	Leucine-rich repeat receptor-like protein kinase (LRR-RLK)	*FON1*, *FLORAL ORGAN NUMBER1*	Os06g0717200	*AtCLV-1*, *CLAVATA1*	At1g75820	receptor protein kinase
* fea2*, *fasciated ear2*	GRMZM2G104925	LRR family protein	*fea2*, *fasciated ear2*	Os02g0603100	*AtCLV2*, *CLAVATA2*	At1g65380	LRR family protein
* msca1*, *male sterile converted anther1*	GRMZM2G442791	glutaredoxin	*MIL1*, *MICROSPORE-LESS1*,	Os07g05630	No homology		
No homology					*SPL/NZZ*, *SPOROCYTE-LESS*	At4g27330	a putative transcription factor
2. Abaxial/Adaxial patterning of anthers							
* mwp1*, *milkweed pood1*	GRMZM2G082264	Myb-like DNA-binding domain	*RL9*, *ROLLED LEAF9*	Os09g0395300	*KAN1*, *KANADI1*	At5g16560	Homeodomain containing superfamily protein
	GRMZM2G480903	Glutaredoxin C8	Grx-C9	Os04g32300	*AtROXY1*	At3g02000	glutaredoxin-C7
	GRMZM2G470756	Glutaredoxin C8	OsGrx_C8	Os02g30850	*AtROXY2*	At5g14070	Thioredoxin superfamily protein
	GRMZM2G030877	bZIP transcription factor		Os11g05480	*AtTGA9*	At1g08320	bZIP transcription factor
	GRMZM2G006578	bZIP transcription factor		Os09g0489500	*AtTGA10*	At5g06839	bZIP transcription factor
	GRMZM2G067205	C2H2 zinc finger protein	*OsJAG*, *SL1*, *STAMENLESS1*	Os01g03840	*AtJAG*, *JAGGED*	At1g68480	zinc finger transcription factor
	GRMZM2G088112			Os01g03840	*AtJAG*, *JAGGED*	At1g68480	zinc finger transcription factor
	GRMZM2G088112			Os01g03840	*At NUB*, *NUBBIN*	At1g13400	zinc finger transcription factor
* yab10*, *yabby homolog 10*	GRMZM2G167824			Os10g36420	*AtYABBY1 (AFO,FIL)*	At2g45190	transcription factor
	GRMZM2G145201	RNA dependent RNA polymerase	*SHL2*, *SHOOTLESS2*	Os01g0527600	*RDR6*, *SDE1*, *SGS2*	At3g49500	RNA-dependent RNA polymerase 6
	GRMZM5G809695	LRR receptor-like protein kinase		Os06g0203800	*ER ERECTA*	At2g26330	LRR receptor-like serine/threonine-protein kinase
	GRMZM2G463904	LRR receptor-like protein kinase		Os06g0130100	*ERL1 ERECTA-like1*	At5g62230	receptor-like protein kinase
	GRMZM2G082855	receptor-like protein kinase		Os06g0203800	*ERL2*, *ERECTA-like2*	At5g07180	receptor-like protein kinase
* rld1*, *rolled leaf1*	GRMZM2G109987	bZIP transcription factor		Os03g0109400	*REV*, *REVOLUTA*	At5g60690	homeobox-leucine zipper protein
* rld2*, *rolled leaf2*							
	GRMZM2G053987	Mitogen-activated protein kinase		Os03g17700	*MPK3*	At3g45640	Mitogen-Activated Protein Kinase
	GRMZM2G002100	Protein tyrosine kinase		Os06g06090	*MPK6*, *MAPK6*	At2g43790	Mitogen-Activated Protein Kinase
3. Anther cell layer differentiation							
* am1*, ameiotic1*	GRMZM5G883855	Protein with coiled-coil domain	*OsAM1*,	Os03g44760	*SWI1 /DYAD*, *SWITCH1*	At5g51330	
* mac1*, multiple archesporial cells1*	GRMZM2G027522	Small secreted protein	*TDL1A*, *TAPETUM DETERMINANT-LIKE1A*	Os12g28750	*TPD1*, *TAPETUM DETERMINANT*	At4g24972	
	GRMZM2G447447	LRR receptor-like protein tyrosine kinase	*MSP1*, *MULTIPLE SPOROCYTES1*	Os01g0917500	*EMS1/EXS*, *EXCESS MICROSPORO-CYTES1*	At5g07280	leucine-rich repeat receptor kinase
	GRMZM2G107484	LRR protein tyrosine kinase		Os01g0917500	*EMS1/EXS*, *EXCESS MICROSPOROCYTES1*	At5g07280	LRR transmembrane protein kinase
* Zmserk1*, *somatic embryogenesis receptor-like kinase1*	GRMZM5G870959	LRR receptor-like protein kinase	*BRASSINO STEROID INSENSITIVE1*	Os4g0457800	*SERK1*, *SOMATIC EMBRYOGENESIS RECEPTOR KINASE1*	At1g71830	receptor-like kinase
* Zmserk2*, *somatic embryogenesis receptor-like kinase2*	GRMZM2G115420	LRR receptor-like protein kinase			*SERK1*, *SOMATIC EMBRYOGENESIS RECEPTOR KINASE1*	At1g71830	receptor-like kinase
	GRMZM2G150024	LRR receptor-like protein kinase		Os08g0174700	*SERK2*, *SOMATIC EMBRYOGENESIS RECEPTOR KINASE2*	At1g34210	LRR receptor-like protein kinase
	GRMZM2G141517	LRR receptor-like protein kinase		Os07g0134200	*At BAM1*, *BARELY ANY MERISTEM1*	At5g65700	LRR receptor-like protein kinase
	GRMZM2G043584	LRR receptor-like protein kinase		Os03g0228800	*At BAM2*, *BARELY ANY MERISTEM2*	At3g49670	LRR receptor-like protein kinase
	GRMZM2G017409	LRR receptor-like protein kinase		Os07g0602700	*AtRPK2*, *RECEPTOR-LIKE PROTEIN KINASE2*	At3g02130	receptor-like protein kinase
* ocl4*, outer cell layer4*	GRMZM2G123140	HD-ZIP IV transcription factor		Os10g0575600	*HB-7*	At5g46880	homeobox-leucine zipper protein HDG5
	GRMZM2G163233	bHLH transcription factor	*UDT1*, *Undeveloped Tapetum*	Os07g0549600	*DYT1*, *DYSFUNCTIONAL TAPETUM1*	At4g21330	bHLH transcription factor
4. Maintenance of cell layer identity							
* male sterile 45*, ms45*	GRMZM2G307906	Strictosidine synthase		Os03g15700		At2g32600	
	GRMZM2G139372	bHLH transcription factor	*TDR*, *TAPETUM DEGENERATION RETARDATION*	Os02g0120500	*AMS*, *ABORTED MICROSPORES*	At2g16910	bHLH transcription factor
	GRMZM5G890224		*PTC1*, *PERSISTANT TAPETAL CELL1*	Os09g0449000	*AtMS1*, *MALE STERILE1*	At5g22260	PHD-type transcription factor
	GRMZM2G120987	NAD-dependent epimerase/dehydratase	*DPW*, *DEFECTIVE POLLEN WALL*	Os03g0167600	*MS2*, *MALE STERILE2*	At3g11980	fatty acyl-CoA reductase
	GRMZM2G476652			Os07g0609766	*LFR*, *LEAF AND FLOWER RELATED*	At3g22990	ARM repeat superfamily protein
	GRMZM2G408897			Os03g0716200	*MMD1*, *MALE MEIOCYTE DEATH1*	At1g66170	PHD finger protein
	GRMZM2G308034	MYB family transcription factor		Os03g0296000	*TDF1*, *DEFECTIVE IN TAPETAL DEVELOPMENT AND FUNCTION*, *MYB35*	At3g28470	R3 MYB transcription factor

LRR, leucine-rich repeat; NAD, nicotinamide adenine dinucleotide; SRF, serum response factor.

## Discussion

The value of well-constructed genetic screens is immense, because a comprehensive list of genes involved in a complex process provides multiple entry points for further analysis, particularly when an allelic series is available for a locus. Maize anther development requires approximately one month, from primordium inception through pollen shed. Our focus was on the early events during this interval, the period of initial anther formation into a four lobed structure, cell fate specification, rapid cell proliferation, acquisition of cell-type specific differentiation, and maintenance of these special characteristics through successful initiation of meiosis. To date, most maize male sterile mutants exhibit post-meiotic defects (Skibbe and Schnable 2005), and this was also the result in this screen: about 10% (29 mutants/244 lines screened or 25 loci/244 lines) of the presumptively male−sterile lines screened exhibited phenotypes in early anther development. One explanation for the lack of proportional representation of premeiotic mutants could be that many genes involved in controlling anther cell fate, proliferation, and expansion are also required in earlier steps in the lifecycle. It is a fact of life that the genetics of floral development is restricted to studying those genes with little or no impact earlier in the life cycle. Pollen development, on the other hand, involves expression of many genes (Ma *et al.* 2008) that are not expressed in young anthers or in leaves. Mutations in these genes would thus be expected to result in viable plants with normal flowers bearing normal anthers containing defective pollen.

Using the collection of historic and newly identified loci involved in anther lobe cell fate specification and differentiation, we can now recognize four categories of defects and provide microscopic evidence and allelism test data to define distinct loci within each of these categories. With this categorization it is clear that successive steps in anther development each require multiple genes. Within each layer, cells divide anticlinally and expand in stereotyped patterns to add girth and length to the growing anther (Kelliher and Walbot 2011), but overall growth is not coordinated by a meristem or any detectable gradient with an anther. Instead, there are local controls visualized as patches of cells synthesizing DNA coordinately and local structural constraints that keep the middle layer and tapetal cells aligned after periclinal division of the secondary parietal layer despite differences in cell division frequencies (Kelliher and Walbot 2011). The many cases of multiple defects, particularly the cases in which one layer fails followed by consequences in other locular layers support the concept that there are complex signaling networks coordinating growth and differentiation within and between the layers. This facet of local growth controls within maize anthers is paralleled by observations in Arabidopsis sepal epidermis in which as yet undefined local growth controls operate to result in continued cell division in some zones *vs.* polyploidy and substantial cell expansion in neighboring patches (Roeder *et al.* 2010).

### Control of anther identity

The initiation of anther development begins as stamen primordia emerge from the floral meristem. Only two of 244 mutant lines lacking anthers in spikelets were found in the screen. Stamen organ identity in Arabidopsis is conferred by combined action of APETALA (AP3), PISTILLATA (PI), SEPALLATA1-4 (SEP1-4), and AG (reviewed by Ma 2005 and Chang *et al.* 2011). In maize, stamen organ identity is regulated by the AP3 ortholog SILKY1 (SI1), by putative PI orthologs Zmm16 and Zmm29, as well as by AG homologs Zmm2, ZAG1-ZAG3 (Whipple *et al.* 2004). In *silky1* mutant plants, stamens are converted to carpels (Ambrose *et al.* 2000). AG, a plant-specific transcription factor, is activated by WUSCHEL in the presence of LEAFY (LFY) to generate the stamen primordium (Lenhard *et al.* 2001; Ikeda *et al.* 2009). *LFY* is an ortholog of the meristem identity gene *FLORICAULA* from *Antirrhinum*, plays an important role in the reproductive transition by establishing the expression of ABC floral organ identity genes (Weigel *et al.* 1992; Weigel and Meyerowitz 1994). Mutations in the maize duplicate *FLORICAULA/LFY* orthologs, *zfl1* and *zfl2*, cause disruption of floral organ identity and patterning, as well as defects in inflorescence architecture; no stamens or two abnormal twisting stamens develop in male spikelets of double *zfl1 zfl2* mutant (Bomblies *et al.* 2003) suggesting a role of these genes in maize anther development. WUSCHEL is also known to control the stem cell activity of the Arabidopsis floral meristem by antagonistic activities with CLAVATAs (CLVs) (Bhalla and Singh 2006). Interestingly, maize orthologs of *CLV1*, *thick tassel dwarf1* (*td1)*, and *CLV2*, *fasciated ear2* (*fea2*; Table3) exhibit extra anthers (Bommert *et al.* 2005; Taguchi-Shiobara *et al.* 2001). Lack of anthers in ms-si*355 and ems*71990 suggests that these genes can be involved in any step of anther identity control.

AG also activates expression of *SPL/NZZ*, a key regulator of anther identity and currently the first gene involved in anther cell fate specification in Arabidopsis (Ito *et al.* 2004). In maize, however, the first defined step is defined by the glutaredoxin encoded by *msca1*, not by a transcription factor. Early anther lobes are composed of equivalent, multipotent L2-d cells and any of them can acquire an AR fate. AR cell specification is determined by redox status (Kelliher and Walbot 2012). It has been proposed that MSCA1-mediated events are triggered by hypoxic conditions arising naturally within growing anther tissue (Kelliher and Walbot 2012). During the screen, we identified two novel alleles of maize *msca1* (Figures 2D, F). Like *spl/nzz* mutants, *msca1* mutants do not form AR cells, anther wall layers do not develop and locules are filled with parenchyma-like cells. In contrast to *spl/nzz* mutants, nonfunctional vascular strands are present in each lobe of *msca1* anther and stomata are present in the epidermis (Chaubal *et al.* 2003); neither structure is present in normal maize anthers. BLAST and phylogenetic analyses did not identify a *msca1* orthologous gene in Arabidopsis despite the large number of GRX genes in this species (Figure 9). An orthologous gene was found in rice, Os07g05630 (Table 3). An insertion in this gene was recently discovered in a rice spontaneous male sterile mutant *microsporeless1* (*mil1*; Hong *et al.* 2012). Like *msca1*, *MIL1* encodes a plant-specific CC-type glutaredoxin; mutations in *MIL1* result in anther lobes that lack microspores and normal wall layer cell types. However, the *mil1* rice mutant shows defects later in anther development than the early step disrupted by *msca1* in maize. In *mil1*, sporogenous cells appear to be specified normally, but subsequent steps fail. No vascular strands or stomata were reported for the rice *mil1* mutant.

### Abaxial/Adaxial patterning of anthers

Once plant organs initiate as a bulge at the flank of a meristem, growth occurs in three different directions: proximal−distal, abaxial−adaxial, and medial−lateral axes. Organs elongate along the proximal-distal axis. The surface of the organ facing the meristem is the adaxial surface, while the organ surface facing away from the meristem is the abaxial surface. In a developing spikelet, the proximal (nearer the meristem) filament connects the distal anther to the plant body; the two anther lobes facing the meristem are the adaxial lobes whereas the other two lobes are abaxial. In anthers from both mutants of the allelic pair *vlo1-ems71924* and *vlo1-ems72032*, the abaxial lobes develop properly with all wall layers, while the adaxial lobes fail to form (Figure 3, B−C), suggesting defects in abaxial-adaxial polarity. Maize mutants with defects in abaxial/adaxial patterning of leaves have been isolated previously (Timmermans *et al.* 1998; Juarez *et al.* 2004; Candela *et al.* 2008). These studies elucidated the mechanisms of regulation of adaxial-abaxial identity in leaf development (reviewed by Husbands *et al.* 2009) whereas mechanisms underlying establishment of adaxia−abaxial polarity in stamens remain largely unknown. Although the stamen is morphologically different from the leaf, it may be modified leaf because stamens are considered to have evolved from sporangium-bearing leaves (sporophylls) (reviewed by Feng and Dickinson 2010a). It is not clear to what extent mechanisms established in modern leaves are applicable to anthers.

In Arabidopsis, the *roxy1 roxy2* double mutant, *tga9 tga10* double mutant, and several other mutants including *jagged* (*jag*) and *wus1* also exhibit two-lobed anthers. The *TGA9* and *TGA10* genes encode basic leucine-zipper transcription factors that are activated by glutaredoxins ROXY1 and ROXY2; plants lacking TGA9 and TGA10 have defects similar to those in *roxy1 roxy2* double mutants (Murmu *et al.* 2010). *JAGGED* encodes a putative zinc finger transcription factor required for proper lateral organ shape. Together with *NUBBIN*, it is involved in both stamen and carpel development (Dinneny *et al.* 2006). The leucine-rich receptor-like protein kinases ERECTA (ER) and ER-Like1 and 2 (ERL1, 2) as well as the mitogen-activated protein kinases MPK3 and MPK6 also were shown to be important for proper anther lobe formation (Hord *et al.* 2008). However, only triple mutants (*er105*, *erl1-2*, *erl2-1*) fail to form one or more of the four anther lobes; none of the single mutants causes a severe anther phenotype. In rice, a mutation in *SHOOTLESS2* (*SHL2*) causes defects in the establishment of anther adaxial/abaxial polarity (Toriba *et al.* 2010). *SHL2* encodes an RNA-dependent polymerase that is involved in posttranscriptional gene silencing. Further studies on the *vlo1* maize mutant, including cloning this, gene will help us to understand whether it defines a novel step in the abaxial-adaxial patterning of anthers in maize.

### Anther wall layer differentiation

Intercellular signaling pathways play significant roles in cell-cell communication required for plant organ development. Locally acting signals and receptors regulate anther wall layer patterning in Arabidopsis (reviewed by Zhao 2009), rice (Zhang *et al.* 2011), and probably in maize. The membrane-localized leucine-rich-repeat receptor-like kinases EMS1/EXS in Arabidopsis and MSP1 in rice were shown to interact with their corresponding ligand TPD1 or TDL1A respectively (Jia *et al.* 2008; Zhao *et al.* 2008).

The maize ortholog of rice *TDL1A*, *mac1*, encoding a small secreted protein not only suppresses AR cell proliferation, but also promotes periclinal division in the adjacent L2-d cells (Wang *et al.* 2012). *mac1* mutant anthers contain excess AR cells but lack the tapetal and the middle layers. It has been speculated that MAC1 may play dual roles by binding to different receptor kinases in the AR cells and L2-d cells (Wang *et al.* 2012). To date, receptors with an ability to bind MAC1 have not been isolated in maize. BLASTs of rice *MSP1* mRNA against *Zea mays* B73 Refgen_v2 sequences uncovered the maize putative orthologous gene GRMZM2G447447 (Table 3) located on chromosome 3 between molecular markers IDP3115 and IDP6021. Its predicted product possesses motifs assigned to serine/threonine kinase and phosphorylation activities. Further experiments will be required to determine whether this locus is required for maize anther development and functions as the receptor for the MAC1 ligand.

The newly discovered mutant *ems63089* displays some features of the *mac1* phenotype: absence of tapetal and middle layers (Figure 4, A−C). However, an excess of microsporocytes has not been observed in *ems63089*; instead, the mutant has even fewer microsporocytes than wild type. It is unknown to what extent the phenotypes of mutated alleles of the orthologous genes may vary. Some species-specific differences in signaling pathways are expected, for example, both anther and ovule are affected in rice *msp1* mutant plants (Nonomura *et al.* 2003) whereas only the anther is affected in Arabidopsis *ems1/exs* mutant (Zhao *et al.* 2002). Cloning and further characterization of *ems63089* could define a novel member of a signaling pathway in maize.

### Lose of cell proliferation control

Control of total cell numbers within an anther cell layer requires modulation of anticlinal cell division patterns. Generation of a new cell layer requires cells in the original layer to divide periclinally only one time. Our screening of maize male sterile mutants showed that cell divisions and cell differentiation during anther development are coupled and genetically controlled. Finite numbers of divisions within each cell lineage are essential for fertile anther development. Most striking are mutants with defects in periclinal division control which generate patchy, partial, or complete novel rings of somatic cells (Chaubal *et al.* 2000; Vernoud *et al.* 2009). The identity of these ectopic cells is not clear, and often neighboring layer cells mis-differentiate as well. *mtm99-66* is a novel allele of *ocl4*, which is exclusively expressed in epidermal cells. The transcription factor encoded by this gene plays a role in suppressing additional division in endothecium precursor (Vernoud *et al.* 2009). An additional periclinal division in the middle layer precursor results in five-layered anther wall in *ems72091* (Figure 5, E−H). The wild-type allele of this gene probably controls the number of cells in the middle layer suppressing additional periclinal division. The *ems72091* mutant phenotype is opposite to that of Arabidopsis *receptor-like protein kinase2* (*rpk2*) mutant, which lacks the middle layer. Only three layers surround microsporocytes in the *rpk2* anthers. The RECEPTOR-LIKE PROTEIN KINASE2 promotes the periclinal division and differentiation of middle layer cells from inner secondary parietal cells (Mizuno *et al.* 2007). The maize RPK2 ortholog has not been isolated to date. Although the middle layer has no precisely ascribed functions and is not adjacent to the sporogenous cells, nevertheless middle layer defects result in aborted microgametogenesis and male sterility. We suggest that unknown aspects of cell−cell communication coordinate cell proliferation and differentiation within the entire anther such that defects in one cell type cause organ growth arrest.

Shortly after the periclinal division of the secondary parietal cells, ms*6015 tapetal initials exhibit extra divisions. Typical tapetum morphology has been never observed in this mutant (Figure 6A). “Tapetal” cells divide anticlinally and/or with abnormal (randomized new cell walls) division orientation generating cells that remain uninucleate and lack characteristics of normal tapetum. In *ms23*, the tapetal initials divide precisely once to generate a bilayer in which the cells remain uninucleate and fail to acquire other tapetal characteristics (Chaubal *et al.* 2000). In contrast, in ms32-ms*6066, there are one to two or more extra layers sandwiched between meiocytes and middle layer initials; no cells exhibit any characteristics of maturing tapetal cells. It is possible that in these three mutants, tapetal initials and later tapetal precursors fail to enter terminal differentiation and therefore do not stop dividing. If so, the wild-type alleles of these genes may suppress cell proliferation by triggering terminal differentiation. In contrast, the LRR receptor kinase signaling complexes described in the previous section can stimulate tapetal initial proliferation specifically promoting only periclinal division in L2-d cells. In mutants with disrupted genes, tapetal initials fail to divide and differentiate. Induction of expression of *EMS1* in the few tapetal initials in *ems1* plants can restore both proliferation and differentiation cells into normal tapetal cells (Feng and Dickinson 2010b). Analysis of transgenic lines with restored tapetum in different patterns varying from the normal monolayer to clumps of multilayered tapetum demonstrated that integrity of the tapetal monolayer is crucial for the maintenance of the polarity of divisions within it (Feng and Dickinson 2010b). Exclusively anticlinal divisions of tapetal initials took place if promoter drove *EMS1* transcription to attain an effective threshold before the fragmentation of the monolayer of tapetal initials. A mixture of anti- and periclinal divisions occurred to generate tapetal layering if *EMS1* expression was triggered after the fragmentation of monolayer (Feng and Dickinson 2010b). Spatial and temporal relationships of gene function may also explain the exclusively anticlinal divisions in ms*6015 and one or two periclinal divisions in addition to anticlinal divisions in *ms23* and *ms32*. The tapetum adjacent to the sporogenous cells plays a crucial role in supplying nutrients to microsporocytes and providing their release form tetrads. Therefore most mutations with defects in the tapetum cause male sterility.

### Cell layer degeneration

Cell death occurs in plants but is an uncommon mechanism shaping plant organs and tissues; however, it is a common end point when development goes awry. In many of the mutants described here, aberrant cells are recognizable by their extensive vacuolization, failure to maintain a dark-staining cytoplasm, and lack of cell wall rigidity. It is presently unclear whether abnormal development triggers the general cell death program (Jones 2001; Lam 2004) or whether developmentally abnormal cells die in cell type−specific processes.

Normal anther development includes a temporally coordinated crushing of the middle layer and later tapetal degeneration mediated by the general programmed cell death (PCD) pathway. Although tapetal degeneration occurs in the wild-type tapetum after microspore mitosis 1, the first hallmarks of PCD were observed in tapetum as early as the premeiotic stage (Varnier *et al.* 2005). Decisions about cell death based on the integration of various signals are probably made long before visible signs of cell degradation. In addition to vacuolization, tapetal cell deterioration is marked by cell shrinkage, polarization of cytoplasmic material, thinning of cell walls that become less distinct between adjacent cells, and DNA fragmentation. Several key genes required for the establishment of PCD have been identified (Li *et al.* 2006, 2011; Phan *et al.* 2011). Quantitative reverse-transcription polymerase chain reaction analysis showed that most genes implicated in PCD are up-regulated as anthers mature (Skibbe *et al.* 2008). Failure to properly regulate cell death results in plant sterility: both premature cell layer degradation and abolition of the tapetum suicide program lead to microspore abortion (Kawanabe *et al.* 2006; Vizcay-Barrena and Wilson 2006; Shi *et al.* 2009). The Arabidopsis *MALE STERILITY 1* (*MS1*) and *ABORTED MICROSPPORES* (*AMS*) and the respective rice orthologs *PERSISTENT TAPETAL CELL1* (*PTC1*) and *TAPETUM DEGENERATION RETARDATION* (*TDR*; Table 3) control programmed tapetum degeneration. Mutations in *MS1* and *PTC1* encoding PHD-finger protein as well as in *AMS* and *TDR* encoding bHLH transcription factor display delayed tapetum degeneration and lack of tapetal DNA fragmentation (Sorensen *et al.* 2003; Li *et al.* 2006, 2011; Ito *et al.* 2007). Tapetal cells are abnormally vacuolated and enlarged in many mutants that display their defects at the late stages (during or after meiosis). Sometimes the middle layer and endothecium become vacuolated as well. Microspore death may also be caused by poor nutrition or defects in pollen coatings secreted by tapetal cells. It is unclear if a PCD signal can also be conveyed from the microspore toward the peripheral cell layers when meiosis fails.

### Functional failure

Functional failure is an inability to perform a cell type regular function due to low synthetic or metabolic level of some components required for normal plant development. Formation of callose walls in prophase meiocytes is a characteristic feature of normally developing anthers (Abramova *et al.* 2003). Callose is essential for sequestering the PMCs from each other and from tapetum. Too little or too much callose is associated with degeneration of developing microspores and plant sterility (Chen and Kim 2009; Wang *et al.* 2010). Callose dissolution is under strict regulation in anther development. Callase is secreted from the tapetal cells to degrade callose and to release microspores from tetrads. The newly detected maize mutant *csmd1* (Wang *et al.* 2011), the historic mutants *ms10*, *ms8* (Wang *et al.* 2010) and its new allele *ms8-mtm99-56*, as well as both newly identified alleles of *ms45*, *ms45-msN2499*, and *ms45-ems6440*, show impaired patterns of callose deposition.

## Conclusion

Although some newly documented maize mutants illustrate a phenotypic and probably functional conservation of mutated genes compared to their orthologs in rice and Arabidopsis, most maize mutants had distinctive phenotypes representing the divergence between monocots and eudicots and between rice and maize during higher plant evolution. The screen of nearly 250 maize male sterile mutants has yielded cases with new types of anther failure and permitted definition of four classes of pre-meiotic defects. As the genes corresponding to each mutant are cloned, a more sophisticated comparison of the steps in eudicot and grass anther development can be conducted. The screen and accompanying allelism tests are an essential first step in elucidating loci for future analysis of the evolutionary context of developmental regulation.
